# Preparation and Evaluation of Soft Gellan Gum Gel Containing Paracetamol

**DOI:** 10.4103/0250-474X.54273

**Published:** 2009

**Authors:** M. C. Gohel, R. K. Parikh, S. A. Nagori, S. N. Shah, M. R. Dabhi

**Affiliations:** Department of Pharmaceutics and Pharmaceutical Technology, L. M. College of Pharmacy, Navrangpura, Ahmedabad-380 009, India

**Keywords:** Soft gellan gum gel, gellan gum, paracetamol, dissolution study, melt-in-mouth gel

## Abstract

The objective of this study was to develop soft paracetamol gel using gellan gum as a gelling agent and sodium citrate as a source of cation. Different batches were prepared using three different concentrations of gellan gum (0.1, 0.3, and 0.5%), each with two different sodium citrate concentrations (0.3 and 0.5%). The consistency of the paracetamol gel was dependent on the concentration of gellan gum, sodium citrate and co-solute. The results of dissolution study of soft gel containing 0.3% gellan gum and 0.3% sodium citrate revealed that paracetamol was completely released in 30 min. Polyethylene glycol 400 worked as a solubilizer for paracetamol. All the gels possessed acceptable sensory characteristics when evaluated by human volunteers. Short term stability study carried out for four weeks at different temperatures revealed no considerable changes in performance characteristics of developed optimized formulation.

Gels are formed by aggregation of polymers with minimum two components; the gelling agent and the fluid component. Gellan gum, carrageenan, pectin, sodium alginate and gelatin are widely used gelling agents in pharmaceutical industries. Gellan gum was selected as a gelling agent in the present investigation to prepare easy to swallow oral medicated ready to use soft gel. Gellan gum is a high molecular weight water-soluble linear anionic polysaccharide produced by the fermentation of the organism *Sphingomonas elodea*[1]. The native form of gellan gum contains two acyl substituents, namely acetate and glycerate. Acylated gellan gum gives soft, very elastic and non-brittle gels whereas deacylated gellan gum gives hard, non-elastic and brittle gels under optimum gelling conditions. As deacylated gellan gum forms brittle gels with weak gel network, they crumble in mouth to cleverly mimic the ‘melt in mouth’ sensation with release of water and associated flavors, which helps in easy release of water soluble drug from the gel dosage form. Free carboxylate groups are present in the structure of gellan gum; therefore gellan gum is anionic in nature and thus it would undergo ionic gelation in the presence of cations such as Ca^++^, Mg^++^, K^+^, Na^+^ and H^+^ from acid[2]. The mechanism of gelation involves the formation of double helical junction zones from random coil chains (coil-to-helix transition) followed by aggregation of double helical segments to form a three-dimensional network by complexation with cations and hydrogen bonding with water[3]. Aggregation behavior is affected by the pH of the solution[4]. By varying the concentration of gellan gum and cation, gels with different gel strength and gel texture can be manufactured.

The objective of this investigation was to develop hydrophilic gel dosage form for oral administration of paracetamol. This gel dosage form is suitable for pediatric, geriatric patients or patients with dysphagia. The gel dosage form can be swallowed easily without water. The patient will not experience chocking of throat, as the gels are soft and smooth. The gel dosage form outpasses the liquid dosage form from the viewpoints of patient acceptance and an attractive appearance. The problem of dose measurement by patients is outweighed as oral medicated gels are to be packed in unit dose. The gel dosage form can be versatile in nature in the sense that it can be used as such or it can be taken with food items such as biscuits and breads.

Paracetamol, an analgesic, antipyretic agent, a BCS (biopharmaceutical classification system) class III drug, was selected as a model[5]. Paracetamol tastes bitter and hence taste masking is made one of the objectives in developing the paracetamol soft gel.

Kelcogel^®^ (dry gellan gum powder) was kindly gifted form CP Kelco (USA). Paracetamol IP was gifted by Green Pharmaceuticals (India). Polyethylene glycol 400 (PEG 400) and citric acid was procured from Laser Laboratories (India). Methylparaben and propylparaben were procured from Apex Pharmaceuticals (India). Sucralose and sodium citrate were kindly gifted from Lincoln Pharmaceuticals Ltd. (India). Food grade sucrose was procured from the local market.

Dry gellan gum powder was dispersed in 50 ml of distilled water maintained at 95°. The dispersion was stirred at 95° for 20 min using a magnetic stirrer (Remi Magnetic Stirrer 2MLH, Mumbai, India) to facilitate hydration of gellan gum. The required amounts of co-solutes (sucrose and sucralose) were added to the gellan gum solution with continuous stirring and the temperature was maintained above 80°. Paracetamol, PEG 400, citric acid and preservatives (methylparaben, propyleparaben) were added with stirring. Finally, required amount of sodium citrate was dissolved in 10 ml of distilled water and added to the mixture. The weight of the gel was monitored continuously during manufacturing and finally it was adjusted to the 100 g with distilled water. The mixture containing gellan gum, paracetamol and other additives was packed in polyethylene bag with airtight seal. The mixture was allowed to cool to room temperature (25±5°) to form gel. The gels were prepared using three different concentrations of gellan gum (0.1, 0.3, and 0.5%), each with two different sodium citrate concentrations (0.3 and 0.5%). The composition of paracetamol soft gel (batches PG1-PG6) is shown in Table 1.

**TABLE 1:** FORMULATION OF PARACETAMOL SOFT GEL

Ingredients	Batch Code					
	
	PG1	PG2	PG3	PG4	PG5	PG6
Paracetamol %	2.5	2.5	2.5	2.5	2.5	2.5
Gellan gum %	0.1	0.1	0.3	0.3	0.5	0.5
PEG 400%	10	10	10	10	10	10
Citric acid %	0.05	0.05	0.05	0.05	0.05	0.05
Sucrose %	66	66	66	66	66	66
Sucralose %	0.3	0.3	0.3	0.3	0.3	0.3
Sodium citrate %	0.3	0.5	0.3	0.5	0.3	0.5
Methylparaben (mg)	0.18	0.18	0.18	0.18	0.18	0.18
Propylparaben (mg)	0.02	0.02	0.02	0.02	0.02	0.02
Raspberry flavor %	2	2	2	2	2	2
Water %, up to	100	100	100	100	100	100

The paracetamol soft gels were examined for appearance in terms of clarity, texture and consistency. Paracetamol soft gels were also evaluated for viscosity, pH, drug content and *in vitro* drug release.

Texture of the soft gel in terms of stickiness and grittiness was evaluated by visual inspection of the product after mildly rubbing the gel sample between two fingers. Viscosity of batches PG1-PG6 was measured using Brookfield DV-II+Pro viscometer. The paracetamol soft gel was squeezed out from the polyethylene plastic bag by making a cut of uniform size on the bag and viscosity was measured using spindle number LV4 at the rotation of 50 RPM at 25±1°. The viscosity measurements were made in triplicate using fresh samples each time. The results of the viscosity measurement of the soft gel (batches PG1-PG6) are shown in Table 2. The pH of paracetamol soft gel was measured using Electroquip Digital pH meter at 25±1°. The pH of the soft gels is shown in Table 2. Drug content of the paracetamol gel was estimated by eluting the drug from 10 g of gel in phosphate buffer pH 5.8. The drug content was estimated spectrophotometrically at 243 nm after filtering the sample through 0.45 μ filters.

**TABLE 2:** EVALUATION OF PARACETAMOL SOFT GEL

Parameters	Batch Code (n=3)					
	
	PG1	PG2	PG3	PG4	PG5	PG6
Viscosity (cPs)	1872±35	2564±52	6575±80	7570±91	10162±107	12182±135
pH	5.93±0.05	6.08±0.08	6.01±0.04	6.12±0.09	5.94±0.05	6.10±0.06

*In vitro* drug release studies was carried out using USP test dissolution apparatus II employing paddle at a speed of 100 RPM using 900 ml of pH 5.8 phosphate buffer as dissolution media at 37±2°. The ready to use soft gel (10 g) containing 250 mg of paracetamol was used in the dissolution test. Five milliliter samples were withdrawn at different time interval and analyzed spectrophotometrically at 243 nm employing Shimadzu-1700 UV/Vis spectrophotometer after suitable dilution of the samples. The fresh dissolution medium was replaced after each withdrawal. The release profile of selected batch PG3 is shown in fig. 1.

**Fig. 1:**
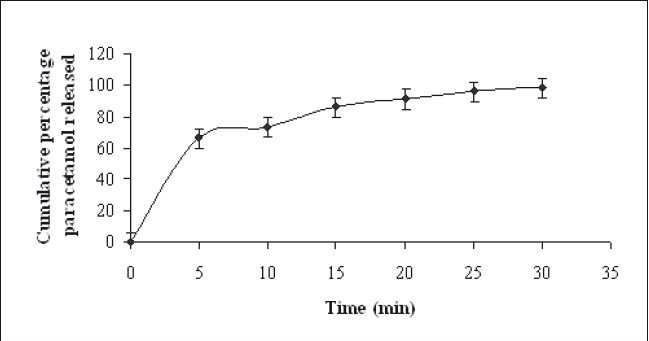
Release profile of paracetamol soft gel PG3. Release profile of paracetamol soft gel batch PG3 (-♦-) in pH 5.8 phosphate buffer.

Ten healthy, adult human volunteers participated in taste evaluation of paracetamol soft gel (PG3). One dose of the paracetamol soft gel (10 g) containing 250 mg of paracetamol was given to every volunteer and they were told to keep the gel in mouth for 5 sec. The volunteers were instructed not to swallow the gel. An interval of 0.5 h was kept for the volunteers between the taste evaluations of both the samples. The volunteers were asked to comment on the bitterness, aftertaste, sweetness and flavor of the gel. Mouth feel in terms of grittiness was also checked. Bitterness and aftertaste were graded from non-bitter (NB) to less bitter (LB) to bitter (BT) to very bitter (VB). Sweetness was graded from less sweet (LS) to sweet (SW) to very sweet (VS). Flavor was assessed from less (LS) to moderate (MD) to good (GD). Mouth feel was assessed from less (TL) to moderate (TT) to good (TS). The results of taste evaluation of paracetamol soft gel are shown in Table 3, respectively.

**TABLE 3:** TASTE EVALUATION OF PARACETAMOL SOFT GEL (BATCH PG3)

Parameters	Volunteers									
	
	1	2	3	4	5	6	7	8	9	10
Bitterness	NB	NB	NB	NB	NB	NB	NB	NB	NB	NB
Aftertaste	NB	NB	NB	BT	NB	NB	NB	NB	NB	NB
Sweetness	VS	VS	VS	VS	VS	VS	VS	VS	SW	VS
Flavor	MD	GD	GD	MD	GD	MD	GD	GD	GD	MD
Mouth feel	TS	TS	TS	TT	TS	TS	TS	TT	TT	MD

NB, BT, SW and VS represent non-bitter, bitter, sweet and very sweet taste. MD and GD represents moderate and good flavor. TT and TS represents moderate and good mouth feel.

The ready to use soft gel (batch PG3) was chosen for short-term stability study. The sample was kept at different temperatures (0-8°, 25±5°, 45±2°) for four weeks. The sample was observed for pH, viscosity and appearance at the interval of 2 week. The results of the stability studies are shown in Table 4.

**TABLE 4:** STABILITY STUDIES OF THE PARACETAMOL SOFT GEL BATCH PG3

Temperature	0-8° (n=3)		25±5° (n=3)		45±2° (n=3)	
			
Weeks	2	4	2	4	2	4
Viscosity(a±45 cPs)	6454	6656	6505	6498	6001	5154
pH (b±0.06)	6.13	6.12	6.10	6.03	6.01	6.09

All the batches of soft gels were transparent in appearance. The gel of batches PG1, PG2 and PG3 were non-sticky and non-gritty while the gel of batch PG4 was slightly sticky but non-gritty. The gel of batches PG5 and PG6 were sticky and gritty. The results of evaluation of paracetamol soft gel batches are shown in Table 2. The gel of batches PG1 and PG2 exhibited fluid like consistency while the gel of batches PG5 and PG6 were very thick in consistency. The viscosity measurement supported visual inspection results. The viscosity of the batches PG3 and PG4 were acceptable. The consistency and viscosity of the soft gels are related to each other because both are dependent on concentration of gellan gum, sodium citrate and co-solute. Effect of concentration of co-solute (sucrose and sucralose) on the viscosity and consistency of all the batches of the soft gel was same because the co-solutes were used at same level in all the batches. It is clearly observed from the results shown in Table 2 that change in the viscosity and consistency of soft gel is strongly influenced by gellan gum concentration. Batch PG3 consisting of 0.3% gellan gum and 0.3% sodium citrate was considered as an optimum batch considering viscosity and appearance.

The pH of the maximum stability of paracetamol in aqueous phase is in between 5 to 7[6]. It is also reported that the apparent viscosity of gellan gum dispersion can be markedly increased by increase in both pH and cation concentration[3–7]. Therefore, the pH of the formulated gels was adjusted and maintained in between 5 to 7 with help of buffering agents such as citric acid and sodium citrate. Sucrose may crystallize in presence of citric acid on standing[8]. Therefore, the amount of citric acid was kept minimum, i.e. just to adjust to the required pH. Sodium citrate was selected as a salt to contribute cation because it also act as sequestrant, buffering agent and helps in maintaining mechanical property of the gel[8]. The drug content in the formulation containing 0.3% gellan gum and 0.3% of sodium citrate was within limits (99.6±1.56%). As shown in [fig. 1], dissolution studies of the paracetamol soft gel containing 0.3% of gellan gum and 0.3% of sodium citrate showed complete release of the drug within 30 min. Paracetamol is sparingly soluble in water but it is soluble 1 in 20 of boiling water[9]. The gel prepared using water, as vehicle was transparent initially. However, turbidity appears after 30 min due to precipitation of paracetamol. PEG 400 was incorporated as a solubilizing agent in the gellan gum soft gel to prevent the precipitation of paracetamol[10]. The gel dosage form, with solubilized form of paracetamol, might facilitate drug dissolution and absorption of paracetamol.

The results of taste evaluation of the batch PG3 paracetamol gel are shown in Table 3. All the ten volunteers perceived the soft gel as non-bitter. The probable reason is that the gelling agents can lower diffusion of bitter substances from the gel to the taste buds. However, the volunteers reported slight bitter after taste. Addition of flavors and sweeteners is the foremost and simplest approach for taste masking especially in the case of pediatric formulations. This provides taste masked gelled pharmaceutical composition for administration of a relatively large amount of unpleasant tasting medicines. Sucrose was selected as a sweetener in soft gel to mask the taste of paracetamol. Sucrose (66.6%) was not able to mask the bitter taste completely because sugar molecules might have been trapped into the gellan gum gel network. Sucralose was selected as an auxiliary sweetener because it is non-carcinogenic and 300-1000 times sweeter than the sucrose[8]. Raspberry flavor was selected because to certain extent it helps in masking the bitter taste of drug[11].

It is reported that low acylated gellan gum gives non-elastic and brittle gel with a very low concentration such as 0.05%. However, the addition of co-solute above 60% results in the formation of flexible and less aggregated gellan gum gel network due to suppression of aggregation of gellan chains and cause reduction in brittleness of low acylated gellan gum gels[12,13]. In the present study, high concentration of sucrose was used and hence high concentrations of gellan gum (0.3 and 0.5%) were used. Sworn *et al.*, reported that the requirements of cations for gelation of gellan gum would decrease with an increase in the concentration of co-solute[12]. This is one of the possible reasons for getting soft gel of optimum consistency with 0.3% gellan gum and 0.3% sodium citrate.

Syneresis is one of the major problems associated with low acylated gellan gum gels. Mashimo *et al*., reported that in gellan gum gel free water exist even in the gel phase[14]. Syneresis was not noticed at room temperature (25±5°) probably due to binding of free water by co-solute[12]. The results of stability studies, shown in Table 4, indicate no considerable changes in pH, viscosity and appearance of the formulations. Precipitation of paracetamol in the soft gels was not observed in any of the gels. A little syneresis was observed in the samples stored at temperature 45±2°.
